# Accurate Triage of Oncological Patients for Safely Continuing Cancer Therapy During the SARS-CoV-2 Pandemic

**DOI:** 10.3389/fonc.2021.707346

**Published:** 2021-10-14

**Authors:** Cristina Gurizzan, Rebecca Pedersini, Carla Fornaro, Chiara Sardini, Manuel Zamparini, Sara Monteverdi, Valeria Tovazzi, Deborah Cosentini, Alberto Dalla Volta, Alice Baggi, Antonella Turla, Pierluigi Di Mauro, Luigi Lorini, Marta Laganà, Susanna Bianchi, Salvatore Grisanti, Francesca Consoli, Elisabetta Conti, Paolo Bossi, Alfredo Berruti

**Affiliations:** Medical Oncology Unit, Department of Medical and Surgical Specialties, Radiological Sciences, and Public Health, University of Brescia at the Azienda Socio Sanitaria Territoriale (ASST)-Spedali Civili, Brescia, Italy

**Keywords:** SARS-COV-2, COVID-19, pandemic, oncology, anticancer therapy

## Abstract

**Objective:**

To evaluate the efficacy of clinical triage of oncological patients for safe continuation of cancer therapy implemented during the first SARS-CoV-2 outbreak.

**Methods:**

Between 25 February and 21 April 2020, patients attending the Medical Oncology Unit, Spedali Civili Hospital, Brescia (Italy) for cancer therapy underwent triage to identify those with no signs and symptoms suspicious for SARS-CoV-2 infection in which antineoplastic treatment could be continued as scheduled. Triage questions investigated common symptoms (e.g., fever, cough, dyspnea, anosmia, dysgeusia, headache, nasal congestion, conjunctival congestion, sore throat, diarrhea, nausea and vomiting); body temperature and pulse oximetry were also recorded. All patients were followed-up for overt SARS-CoV-2 through to 18^th^ May 2020.

**Results:**

Overall, 1180 patients (median age 65 years) underwent triage during the study period. The most frequent primary malignances were breast (32%), gastrointestinal (18%), and lung (16.5%) cancer. Thirty-one (2.5%) presented with clinically evident SARS-CoV-2 infection and tested positive on nasopharyngeal swab testing and/or radiological imaging. Triage identified 69 (6%) grey zone patients with symptoms suspicious for SARS-CoV-2; 5 (7.2%) subsequently developed symptomatic disease. Neither the symptomatic nor the grey zone patients received their scheduled treatment; instead, they were referred for hospitalization or home quarantine.

**Conclusion:**

Triage of oncological patients at our Unit provided for safe continuation of scheduled cancer treatment in 91.5% of patients during the initial SARS-CoV-2 outbreak.

## Introduction

On December 31^st^, 2019, Chinese health authorities reported an outbreak of pneumonia of unknown etiology in the city of Wuhan (Hubei Province, China), which the Center for Disease Control and Prevention of China identified as a novel coronavirus, classified as SARS-CoV-2; in mid-February it was named COVID-19 (1), the combination of Corona VI-rus D-isease and year of identification, 2019. One month later, on January 31^st^, 2020, the World Health Organization (WHO) issued a public health emergency of international concern, and on March 11^th^ it declared a state of pandemic. Italy was the first European country to be hit and the first cases of SARS-CoV-2 infection were confirmed by the Italian National Institute of Health on January 30^th^, 2020.

The most frequent and serious manifestation of the infection is pneumonia, characterized by fever, cough, dyspnea, and bilateral pulmonary infiltrate at imaging (2). The most common clinical symptoms are fever (99%), fatigue (70%), dry cough (59%), anorexia (40%), myalgia (35%), and dyspnea (31%) (2). Dysgeusia and anosmia are early and sometimes the only symptoms (3). Less common are headache, sore throat, rhinorrhea, and gastrointestinal symptoms (e.g., nausea and diarrhea) (1–4). Asymptomatic infections have often been reported, although their frequency is unknown.

Cancer patients may be more vulnerable to SARS-CoV-2 infection because of their underlying illness and immunosuppressed status due to either the disease itself or the anticancer treatment (chemotherapy, radiotherapy, surgery) (5–8). They may also be at greater risk of SARS-CoV-2-related serious events (intensive care, mechanical ventilation, death) than the general population (9, 10).

Controversy surrounds the impact of cancer therapy on the outcome of patients with SARS-CoV-2 infection. Two small studies from China suggested worse outcomes after chemotherapy, whereas recently published larger trials found no significant effect of cytotoxic drug administration within 4 weeks of a positive SARS-CoV-2 test on infection-associated adverse outcomes and patient mortality (11–13). The same was observed for patients receiving hormonal, targeted, and immune therapies (12).

The European Society of Medical Oncology (ESMO), the American Society of Clinical Oncology (ASCO), the National Comprehensive Cancer Network (NCCN), the Italian Society of Medical Oncology (AIOM), and other societies have issued guidelines on how to mitigate the negative effects of SARS-CoV-2 on the diagnosis and the treatment of cancer patients (14–18). The guidelines recommend categorizing cancer patients into high, medium, or low priority based on the Ontario Health Cancer Care Ontario criteria for planning their management (19). ESMO guidelines recommended that cancer patients requiring hospital admission for cancer treatment be tested for SARS-CoV-2 whenever feasible in the context of available resources, regardless of symptoms or chest radiological findings if considered at high risk of mortality in case of SARS-CoV-2 infection.

Few studies published to date have evaluated the role of a triage system as an initial screen to select cancer patients for scheduled therapy after a negative SARS-CoV-2 test (20, 21). To fill this gap, we explored the efficacy of triage implemented during the early days of the first wave of the pandemic in Italy to screen higher priority patients who could safely continue scheduled treatment and to identify patients at higher risk of SARS-Cov 2-infection for which postponement of treatment would be appropriate.

## Methods

For this retrospective single-center cohort study, the data from consecutive cancer outpatients under treatment at Medical Oncology Unit, ASST Spedali Civili of Brescia, Italy, were reviewed.

During the study period (February 25^th^ to April 21^st^, 2020), the Unit had in place most of the protective measures that were subsequently recognized effective in preventing the transmission of viral infection between patients and healthcare workers (22). For scheduled therapeutic access to the Unit, a telephone contact was made the day before, in this way: all patients with outwardly suspicious SARS-CoV-2 symptoms or positive swab did not access the Unit and were referred to the emergency room or general practitioner. Patients admitted to therapy were triaged by a trained nurse who asked about recent symptoms (fever, cough, dyspnea, unexplained fatigue, anosmia, dysgeusia, headache, nasal congestion, conjunctival congestion, sore throat, diarrhea, nausea and vomiting) and recorded pulse oximetry and body temperature. History was taken about close contact with persons who tested positive for SARS-CoV-2 infection or displayed common symptoms of the disease or recent travel to pandemic areas. The full triage questionnaire is provided in **Supplementary Material 1**.

**Figure 1:**
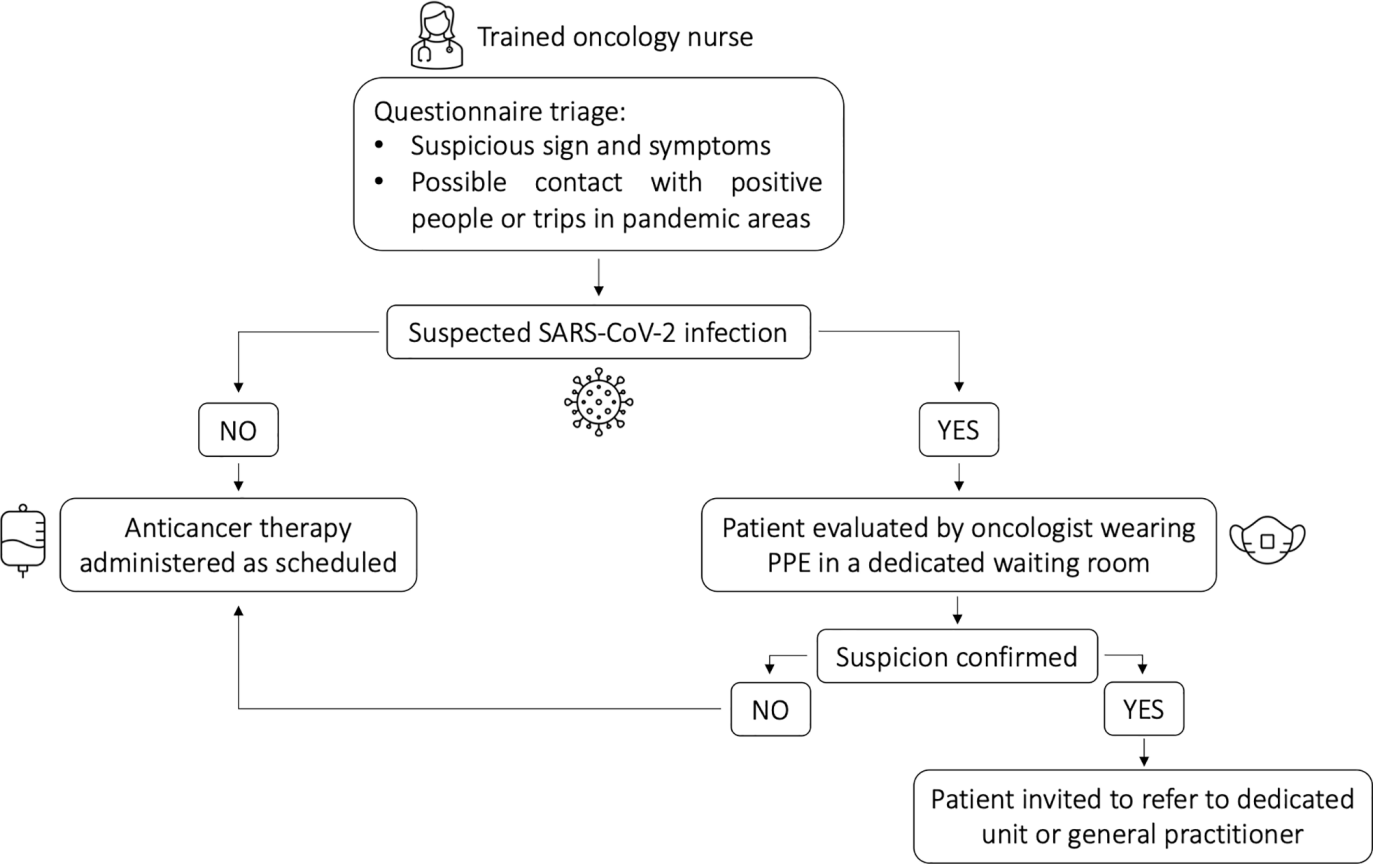
Patient pathway following triage questionnaire.

Triage questions were asked in consequential order according to the presence of a symptom and the suspicion of infection and in this way we created a flowchart by which we selected patients who were eligible for treatment. Patients with signs and symptoms suspicious for SARS-CoV-2 infection were examined by an oncologist wearing personal protective equipment (PPE) in a waiting room separate from the rest of the Unit. If suspicious symptoms and signs of COVID-19 were confirmed, treatment was temporarily discontinued. As nasopharyngeal swab testing was not available at the time of the visit, the patients were referred to preventive quarantine for at least 14 days under the assistance of their general practitioner. Patients who reported close contact with persons who tested positive or had symptoms suspicious for SARS-CoV-2 infection or who had traveled to pandemic areas were also quarantined for at least 14 days and then readmitted for therapy. Otherwise, those who triaged either negative for symptoms or with symptoms of a different cause (e.g., cancer-related, treatment-related, symptoms onset more than 14 days before) continued with their scheduled therapy (**Figure 1**).

These procedures allowed us to select three different patients population: 1) symptomatic SARS-CoV-2 infected patients who were addressed to another unit or their general practitioner, 2) grey zone patients defined as having clinical symptoms (e.g., fever, cough, dyspnea, anosmia, dysgeusia, headache, nasal congestion, conjunctival congestion, sore throat, diarrhea, nausea and vomiting) suspicious for or radiological images suggestive of SARS-CoV-2 infection but had not undergone nasopharyngeal swab testing and 3) patients either asymptomatic or with symptoms of a different cause (e.g., cancer-related, treatment-related, symptoms onset more than 14 days before). The first two groups temporarily interrupted, while the third group continued with the scheduled therapy.

The primary endpoint of this study was to evaluate the effectiveness of triage of cancer patients for continuing their treatment safely as scheduled. The secondary endpoints were: 1) to describe the characteristics and severity of SARS-CoV-2 infection in the patients and the grey zone patients in relation to clinical characteristics of the tumor (stage and type of treatment) and of the patient (age, comorbidities, performance status); 2) to describe the management of the patients with confirmed SARS-CoV-2 infection and the grey zone patients; 3) to estimate the prevalence of symptomatic SARS-CoV-2 infection in the cancer patients under treatment.

### Data Collection and Statistical Analysis

Clinical data, including cancer details, treatment, comorbidities, symptoms reported during triage, and severity of symptoms were extracted. Descriptive statistics were used to analyze the characteristics of the patient cohort. Categorical variables are expressed as frequency and percentage; the distribution of continuous variables was calculated as the mean ± 95% confidence interval (95% CI). Population characteristics are described as the mean (± 95%) CI or percentage.

## Results

A total of 1180 patients were scheduled for treatment at our Unit. Median age was 65 years (range 16-94), 634 (54%) were women and the most frequent primary malignancies were: breast (32%), gastrointestinal (18%), and lung (16.5%) cancer. About half (44.5%) received chemotherapy, 22.5% targeted therapy, and 11.1% immunotherapy. **Table 1** presents the clinical characteristics of the patients. Thirty-one (2.6%) did not keep their appointment because of SARS-CoV-2-related symptoms and positivity for infection on nasopharyngeal swab testing and/or radiological imaging. Testing was performed in the emergency room or organized by the patient’s general practitioner.

**Table 1:** Clinical and demographical characteristics of 1180 enrolled patients.

Patients number (1180)	Characteristics	Number (%)
**Gender**	Female	634 (53.7)
	Male	546 (46.3)
**Age (years)**	Mean 65	
	(range 16 - 94)	
**Performance Status (ECOG)**	0	708 (60)
	1	356 (30.2)
	≥ 2	116 (9.8)
**Comorbidity**	Diabetes mellitus	55 (4.7)
	Hypertension	259 (21.9)
	Cardiovascular events	32 (2.7)
	Chronic respiratory disease	23 (1.9)
	More than one of above	112 (9.5)
	Other comorbidity	238 (20.2)
	None	461 (39.1)
**Primary tumor site**	Breast	373 (31.6)
	Genitourinary system	135 (11.4)
	Lung	195 (16.5)
	Gastroenteric system	215 (18.2)
	Head and neck	51 (4.3)
	Melanoma	112 (9.5)
	Adrenocortical	17 (1.4)
	Neuroendocrine system	37 (3.1)
	Others	45 (3.8)
**Stage**	Early (I-III)	217 (18.4)
	Advanced (IV)	963 (81.6)
**Antineoplastic therapy**	Chemotherapy	524 (44.5)
	Immunotherapy	131 (11.1)
	Target therapy	265 (22.5)
	Hormonotherapy	106 (9)
	Mitotane	13 (1.1)
	Chemotherapy + Target therapy	60 (5.1)
	Chemotherapy + Immunotherapy	16 (1.4)
	CDK4/6 Inhibitors + Hormonotherapy	65 (5.5)

**Figure 2:**
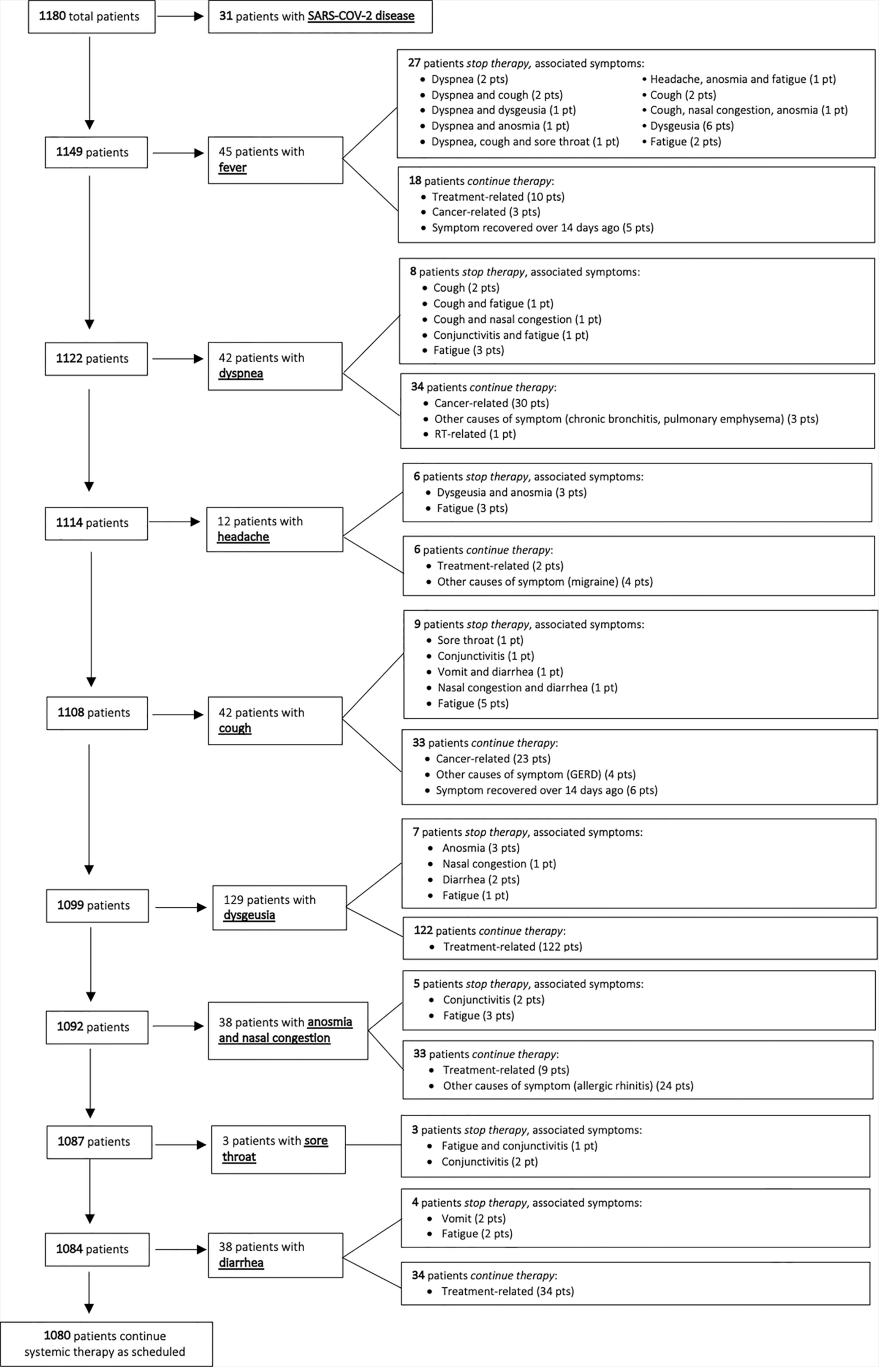
Flowchart of triage questions selecting patients eligible for treatment.

The remaining 1149 patients underwent triage, the complete flowchart is depicted in **Figure 2** and symptoms suggestive of SARS-CoV-2 disclosed by clinical triage are presented in **Table 2**.

**Table 2:** Characteristics of Sars-COV-2 -positive and grey zone patients.

Sars-COV-2 positive		
Patients number (61)	Characteristics	Number (%)
**Gender**	Female	19 (48.7)
	Male	20 (51.3)
**Age (years)**	Median 68	
	Range 31 - 81	
**Performance Status (ECOG)**	0	14 (35.9)
	1	13 (33.3)
	≥2	12 (30.8)
**Comorbidity**	Diabetes mellitus	2 (5.1)
	Hypertension	7 (17.9)
	Cardiovascular events	1 (2.6)
	Chronic respiratory disease	1 (2.6)
	More than one of above	4 (10.2)
	Other comorbidity	10 25.6)
	None	9 (23)
**Primary tumor site**	Breast	12 (30.8)
	Genitourinary system	1 (2.6)
	Lung	10 (25.6)
	Gastroenteric system	5 (12.8)
	Head and neck	3 (7.7)
	Melanoma	2 (6.5)
	Adrenocortical	3 (7.7)
	Neuroendocrine system	0
	Sarcoma	3 (7.7)
**Stage**	Early (I-III)	9 (23)
	Advanced (IV)	30 (77)
**Antineoplastic therapy**	Chemotherapy	21 (53.8)
	Immunotherapy	6 (15.4)
	Target therapy	3 (7.7)
	Hormonotherapy	2 (5.1)
	Mitotane	2 (5.1)
	Chemotherapy + Target therapy	2 (5.1)
	Chemotherapy + Immunotherapy	2 (5.1)
	CDK4/6 Inhibitors + Hormonotherapy	1 (2.6)
**Symptoms**	Fever	27 (69.2)
	Cough	19 (48.7)
	Dyspnea	16 (41)
	Fatigue	13 (33.3)
	Anosmia	4 (10.2)
	Dysgeusia	5 (12.8)
	Headache	4 (10.2)
	Nasal congestion	6 (15.4)
	Conjunctival congestion	0
	Sore throat	2 (5.1)
	Diarrhea	3 (7.7)
	Nausea/Vomit	1 (2.6)
**Management of infection**	Hospitalization	23 (59)
	Quarantine	16 (41)
**Impact of infection on antineoplastic therapy**	Treatment delay	23 (59)
	Treatment withdraw	16 (41)
**Outcome**	Infection resolved	31 (79.5)
	Patient expired	8 (20.5)
**Grey zone patients (without proven infection, i.e. negative swab or not performed)**		
**Patients number (61)**	**Characteristics**	**Number (%)**
**Gender**	Female	30 (49)
	Male	31 (51)
**Age (years)**	Median 68	
	Range 31 - 84	
**Performance Status (ECOG)**	0	20 (32.8)
	1	24 (39.3)
	≥2	17 (27.9)
**Comorbidity**	Diabetes mellitus	3 (5)
	Hypertension	16 (26.2)
	Cardiovascular events	3 (5)
	Chronic respiratory disease	1 (1.6)
	More than one of above	4 (6.5)
	Other comorbidity	13 (21.3)
	None	29 (47.5)
**Primary tumor site**	Breast	12 (19.7)
	Genitourinary system	3 (5)
	Lung	16 (26.2)
	Gastroenteric system	11 (18)
	Head and neck	6 (9.8)
	Melanoma	10 (16.4)
	Adrenocortical	1 (1.6)
	Neuroendocrine system	1 (1.6)
	Other	1 (1.6)
**Stage**	Early (I-III)	13 (21.3)
	Advanced (IV)	48 (78.7)
**Antineoplastic therapy**	Chemotherapy	34 (55.7)
	Immunotherapy	7 (11.4)
	Target therapy	10 (16.3)
	Hormonotherapy	3 (5)
	Mitotane	1 (1.6)
	Chemotherapy + Target therapy	3 (5)
	Chemotherapy + Immunotherapy	3 (5)
	CDK4/6 Inhibitors + Hormonotherapy	0 (0)
**Symptoms suggestive for Sars-COV-2 infection**	Fever	28 (46)
	Cough	21 (34.4)
	Dyspnea	13 (21.3)
	Fatigue	15 (24.6)
	Anosmia	4 (6.5)
	Dysgeusia	6 (9.8)
	Headache	3 (5)
	Nasal congestion	6 (9.8)
	Conjunctival congestion	0
	Sore throat	3 (5)
	Diarrhea	2 (3.3)
	Nausea/Vomit	1 (1.6)

Forty-five patients reported fever (4%); scheduled treatment was postponed in 27 (60%) and they were assigned to at least 14 days of home quarantine. The second triaged symptom was dyspnea (42/1122 patients, 3.7%), however in most cases (34/42, 81%) it was cancer-related and the same was for cough (33/42, 78.6%). Otherwise, therapy was postponed in one half of patients who complained of new onset headache (6/12, 50%).

Dysgeusia was the most frequently reported symptom, however, it was due to chemotherapy in nearly all cases (122/129, 95%) and did necessitate discontinuation of therapy. Anosmia and sore throat were uncommon [10/1092 (0.9%) and 3/1088 (0.3%) patients, respectively] and were associate to postponed treatment due to the coexistence of other suspicious symptoms (i.e., conjunctivitis and fatigue). Nasal congestion and diarrhea (2.6% and 3.5% of cases, respectively) did not necessitate discontinuation of treatment in nearly any case.

Overall, triage identified 69 (5.8%) grey zone patients presenting symptoms strongly suspicious for SARS-COV-2 infection. Complete blood count (CBC) values were available in 45 out of 69 (65%) patients, median neutrophils value was 4.5 x 10ˆ3 (range 0.36-28.01, with normal cutoff values 1.5-8 x 10ˆ3), 4 patients (8.9%) were neutropenic, while neutrophilia was found in 28.9% of cases (13/45). Median lymphocytes value was 1.205 x 10ˆ3 (range 0.32-15.6, with normal cutoff values 0.9-4 x 10ˆ3), with lymphopenia recorded in 35.6% (16/45) of cases, while lymphophilia in just one case (2.2%). More than half of grey zone patients for which CBC was available (26/45, 57.8%) reported a neutrophil-lymphocyte ratio greater than 3.

All grey zone patients were then followed up under quarantine. Fifty-five (80%) underwent nasopharyngeal swab testing in the days following triage and 8 (14.5%) tested positive, 5 of them developing mildly symptomatic disease. The remaining 1080 patients (91.5%) who resulted negative at triage continued their antineoplastic therapy as scheduled (43.5% chemotherapy, 11% immunotherapy, 24.2% targeted therapy, 9.3% hormonotherapy, the remaining 12% a combination of these therapies). None presented symptoms and signs of SARS-COV-2 infection as of May 18^th^ 2020.

## Discussion

Cancer patients under antineoplastic therapy were considered a vulnerable population at the beginning of the SARS-CoV-2 pandemic (23–25). Recent studies, however, have shown that cancer therapy does not lead to more severe coronavirus disease or infection-related death in cancer patients a with positive SARS-CoV-2 test. The present study was conducted during the early months of the pandemic when diagnostic tests for SARS-CoV-2 were not available to screen cancer patients. Our findings demonstrate the feasibility and the efficacy of risk-adapted triage for cancer patients receiving antineoplastic therapy. The stepwise triage we set up allowed for safe continuation of cancer therapies in more than 90% of patients, without interrupting treatment continuity, even during the most critical period of the pandemic and in an area with the highest rate of infection in Italy.

Due to the particularly high infection rates in the Brescia population when the study was conducted, it is likely that many asymptomatic patients who continued scheduled cancer therapy were infected. None underwent nasopharyngeal swab or serological testing. This is a limitation of the present study; however, none who continued their therapy developed symptoms of disease during the follow-up period. These observations are consistent with recent data (12). Only 7% of the grey zone patients subsequently developed symptomatic COVID-19 and only 8 of the 55 patients, who performed the nasopharyngeal swab, tested positive. These data are consistent with the low sensitivity of a triage system implemented to detect SARS-CoV-2 infection, as recently pointed out (20, 21), and confirm that this procedure cannot substitute specific tests (swab, serology).

The triage, therefore, should be considered as a complementary tool to the currently existing molecular or rapid swab tests. A reasonable question is whether our findings are still applicable in the current context. As the pandemic went on, the national health care system improved its organization of services substantially (26–28). Swab screening tests can now be routinely performed in all patients attending our Unit for cancer therapies. However, recent studies showed that, unlike patients with hematological malignancies, patients bearing solid tumors, who receive chemotherapy within 4 weeks of a positive COVID19 test, does not develop a more severe and deadly disease as opposed to the general population (12, 29). Similar results were observed for hormonal therapies, targeted therapies, radiation therapies, and immunotherapies (29). Therefore, oncologists today are less worried than in the past about continuing therapy in cancer patients who test positive for SARS-CoV-2 infection particularly if asymptomatic (29). In this context, a triage, which is set up to distinguish the symptoms potentially attributable to the SARS-CoV-2 infection from those due to the malignant disease or in consequence of the treatments administered, maintains its validity, as it identifies patients who can safely continue oncological treatments, regardless of the outcome of the nasopharyngeal swab. As abovementioned, the triage could also provide complementary information to the molecular nasopharyngeal swab, given that the diagnostic sensitivity of this test is 66-72%, hence it is unable to detect a sizeable portion of infected patients (30). Finally, the results of this study suggest a possible algorithm in which triage is performed as a first approach and only patients who fall into the “grey zone” are addressed to a nasal pharyngeal swab. This procedure deserves to be validated in the future.

The relatively large cohort of patients enrolled in a single centre and the easy reproducibility of our comprehensive clinical triage are the main strengths of the present study. In addition, our triage may be considered as an unexpensive screening tool for cancer patients in countries with lower accessibility to specific SARS-CoV2 screening tests and limited budget. The unavailability of swab and/or serologic tests in all enrolled and triaged patients represents a clear limitation.

In conclusion, our findings indicate that triage for cancer patients is effective to maintain continuity of treatment for most patients. Because of its low sensitivity, triage cannot substitute serological or molecular testing to detect SARS-CoV-2 infection; however, it is a simple test that coupled with currently available molecular or rapid swab tests can aid clinicians in safely selecting patients for cancer therapies. The utility of combining accurate triage with nasopharyngeal swab testing and/or serology is a future research topic of our Institution.

## Data Availability Statement

The datasets presented in this article are not readily available because all data generated or analysed during this study are included in this published article. Requests to access the datasets should be directed to alfredo.berruti@gmail.com.

## Author Contributions

ABe, PB, and RP conceived the study. CG, RP, PB and ABe contributed to manuscript writing. FC, SG revised the manuscript. MZ contributed to the statistical analysis. CG, RP, CF, CS, SM, VT, DC, ADV, ABa, AT, PDM, LL, ML, SB and EC collected data. All authors contributed to the article and approved the submitted version.

## Conflict of Interest

ABe reported advisory board membership or receiving funding unrelated to the submitted study from: Advanced Accelerator Applications, Astellas, Janssen Cilag, Ipsen, Amgen, Novartis, Sanofi. PB reported advisory board membership or receiving funding unrelated to the submitted study from: Merck, Sanofi, Merck Sharp & Dohme, SunPharma, Kyowa Hakko Kirin, Angelini, AstraZeneca, Bristol-Myers Squibb, Helsinn, Novartis, Roche, GSK.

The remaining authors declare that the research was conducted in the absence of any commercial or financial relationships that could be construed as a potential conflict of interest.

## Publisher’s Note

All claims expressed in this article are solely those of the authors and do not necessarily represent those of their affiliated organizations, or those of the publisher, the editors and the reviewers. Any product that may be evaluated in this article, or claim that may be made by its manufacturer, is not guaranteed or endorsed by the publisher.
