# Supplementary material for: Accurate Triage of Oncological Patients for Safely Continuing Cancer Therapy During the SARS-CoV-2 Pandemic
Source: Front Oncol. 2021 Oct 14;11:707346. doi: 10.3389/fonc.2021.707346 (PMC8552044; doi:10.3389/fonc.2021.707346)
Supplement: Supplementary file 1 [file DataSheet_1.doc]

MEDICAL ONCOLOGY COVID 19 TRIAGE MODULE

DATE __________________ MR/MRS/MS_______________________________________

| QUESTION: | **NO** | **YES** | IF YES, SPECIFY: |
| --- | --- | --- | --- |
| Did you swab for COVID 19 positive assessment? |  |  |  |
| Do you currently have fever or have you had fever in the past few days? |  |  |  |
| Do you have cough, a cold or breathing difficulty with shortness of breath? |  |  |  |
| Have you had cough, a cold or shortness of breath with breathlessness in the past two weeks? |  |  |  |
| Have you taken anti-inflammatory drugs such as paracetamol or others in the previous 4 days? |  |  |  |
| Do you have diarrhea or vomiting? |  |  |  |
| Do you have changes in taste and smell? |  |  |  |
| Do you have active conjunctivitis? |  |  |  |
| Are you feeling unwell / tired? |  |  |  |
| Do you have a headache? |  |  |  |
| Do you have joint pain? |  |  |  |
| Have you had any recent cardiovascular events? |  |  |  |
| Did you hang out or do you live with people who swab positive for COVID 19? |  |  |  |
| Did you hang out or do you live with people who have any of the symptoms listed above? |  |  |  |
| Did you hang out or do you live with people who have had contact with positive people for COVID 19? |  |  |  |
| Are you currently working? |  |  |  |
| What work do you do? |  |  |  |

TEMPERATURE DETECTION__________ SATURATION____________________

USER’S SIGNATURE ________________________

OPERATOR'S SIGNATURE ________________________
